# The imbalance of liver resident macrophages polarization promotes chronic autoimmune hepatitis development in mice

**DOI:** 10.7717/peerj.14871

**Published:** 2023-02-07

**Authors:** Gang Chi, Jinhong Pei, Xueqing Li

**Affiliations:** Changzhi Medical College, Changzhi, China

**Keywords:** Autoimmune hepatitis, Kupffer cells, TLR ligands, Polarization imbalance

## Abstract

**Background:**

Autoimmune hepatitis (AIH) is a chronic immune-mediated inflammatory liver disease. At present, it is largely unknown how the innate immune cells influence AIH development.

**Objective:**

To inquiry about mechanism of liver resident macrophages in AIH development, thus offering a new direction for AIH targeted treatment.

**Methods:**

The liver resident macrophages were eliminated by clodronate liposomes in AIH liver tissues, followed by HE and Picrosirius assay to detect liver fibrosis and lymphocyte infiltration. The liver resident macrophages polarization was detected by Immunohistochemistry and qPCR. The collagenase digestion was used to isolate Kupffer cells from AIH mice liver tissues and pro-/anti-inflammatory cytokines were determined by qPCR.

**Results:**

M2 macrophages were the dominant phenotype at early immune response stage and hepatic inflammation was progressively aggravated after depletion of liver resident macrophages. M2 macrophages could effectively delay the development of AIH and could be polarized to M1 macrophages at the disease progresses. TLR2 ligands could promote M2 macrophages producing anti-inflammatory cytokines, whereas TLR4 ligands could promote M1 macrophages producing proinflammatory cytokines. The change of TLR2 and TLR4 ligands could lead to continuous high expression of TLR4 and decreased expression of TLR2 in macrophages to further affect liver resident macrophages polarization state.

**Conclusion:**

TLR2 and TLR4 ligands mediated liver resident macrophages polarization to favor chronic autoimmune hepatitis development.

## Introduction

Autoimmune hepatitis (AIH) is a severe chronic inflammatory liver disease characterized by an ongoing autoimmune reaction directed against hepatic autoantigens (Schultheiß et al., 2021; Mieli-Vergani et al., 2018). However, there is currently no effective treatment for AIH. Most patients respond to steroid therapy and show a favorable outcome, but long-term treatment with steroids may result in unwanted side effects. Most patients almost inevitably relapse when immunosuppression decrease gradually (Schultheiß et al., 2021; Terziroli Beretta-Piccoli, Mieli-Vergani & Vergani, 2022) Thus, the immunological mechanisms underlying AIH progression need to be elucidated to establish more effective therapeutic approaches.

Macrophages are part of the innate immune response to liver injury, and they are functionally categorized into proinflammatory function or anti-inflammatory actions depending on their state of activation (Stone et al., 2019; Czaja, 2015). M1 macrophages are characterized by their pro-inflammatory function and release proinflammatory cytokines to promote inflammation and exacerbate tissue damage (Czaja, 2015; Amin et al., 2006). M2 macrophages are anti-inflammatory and they release anti-inflammatory cytokines (Czaja, 2015; Park et al., 2009). The primary function of macrophages associated with these cytokines production, which is essential for initiating inflammatory response and forming a complex network for immune system homeostasis (Zhang et al., 2018a; Burdo, Lackner & Williams, 2013; Boyman et al., 2007). The liver resident macrophages seem to play a major role in the pathogenesis of AIH. Previous research suggested that activated macrophages were present in portal infiltrates and at sites of interface hepatitis in AIH (Zhang et al., 2018b). Recent studies found M1 macrophages increased, whereas M2 macrophages decreased in AIH patients (Wang et al., 2019). It was reported that hepatic macrophages producing chemokine ligand CXCL9 promote the migration of CD8+ T cells in AIH (Ikeda et al., 2014). The macrophage deletion prevented ConA-induced hepatitis, indicating that immune-mediated liver injury involves macrophage and T cell interactions (Liu et al., 2019). However, the detailed mechanism underlying M1 and M2 macrophage polarization and their change in AIH development remains unclear.

Recent studies showed that TLR2/4-mediated inflammasome activation in CD14+ monocytes was critical to maintaining dysfunctional Tregs in *de novo* autoimmune hepatitis (Arterbery et al., 2018). Moreover, AIH could be related to regulation of TLR4 signaling pathway and the production of proinflammatory cytokines (Wu et al., 2017). TLR4 signal transduction could promote M1 macrophages polarization and TLR2 ligands could drive M2-polarized macrophages in rheumatoid arthritis (Quero et al., 2017; Zhao et al., 2017). M2-polarized macrophages in rheumatoid arthritis patients displayed an impaired anti-inflammatory activity under TLR2 engagement (Quero et al., 2017). TLR2/4 ligand-amplified liver inflammation increased expression of proinflammatory cytokines in AIH (Chi et al., 2018). In this study, the effect of TLR2 and TLR4 ligands on Kupffer cells polarization was explored in AIH development.

## Materials and Methods

### Plamids and *in vivo* gene transfection

Plasmids pCYP2D6, psTLR2 and psTLR4 are expression vectors (pcDNA3.1; Invitrogen, Carlsbad, CA, USA) carrying the cDNA encoding human cytochrome P450 2D6 (CYP2D6), the extracellular domain of murine TLR2 and TLR4, respectively as described previously (Yan et al., 2013; Li et al., 2017). For *in-vivo* gene transfection, plasmids were prepared and analyzed as described previously (Geng et al., 2006). Mice received the injection of plasmid DNA *via* the tail vein (i.v. injection) using the hydrodynamics-based gene delivery technique, which delivers the transgene mainly into the liver (Geng et al., 2006).

### Animal models and treatment

Female 7–8 weeks old C57BL/6 mice were purchased from the Center of Medical Experimental Animals of Hubei Province (Wuhan, China) for investigation. Animals were housed in a 12:12-h light-dark cycle with free access to food and animal care was supervised by veterinarians and trained animal care staff in animal care facilities. The Changzhi Medical College’s Institutional Animal Care and Use Committee approved all animal studies (No. DW2022073). All animal experiments were performed under S2-conditions in the central animal facility and were carried out in compliance with accepted standards of humane animal care as described in the Guide for the Care and Use of Laboratory Animals (National Research Council of the National Academics, 1996). The euthanasia procedures were in accordance with the AVMA Euthanasia Guidelines. Euthanasia was performed using carbon dioxide suffocation and cessation of breathing and any surviving mice at the conclusion of the experiment were euthanized. To induce hepatic inflammation, the mice were intravenously (i.v.) injected with adenovirus or intraperitoneally (i.p.) injected with clodronate liposomes. Data were collected as previously described (Chi et al., 2020). To express human CYP2D6, the mice received i.v. injection of pCYP2D6 plasmid. To induce autoimmune response and AIH, pCYP2D6 plasmid along with adenovirus was injected into the mice. To block the ligands for TLR2 and TLR4 in the liver, mice received the i.v. injection of plasmids psTLR2/psTLR4. To deplete Kupffer cells, 100 μl of clodronate liposomes (Liposoma BV, Amsterdam, Netherlands) per mouse were administered intraperitoneally every 4 days from d20 after AIH mice model establishment. The protocols for the injection of different agents are shown in Supplemental Figures, and indicated in the corresponding figure legends.

### Isolation and culture of mouse Kupffer cells

A modification method of the type IV collagenase digestion *in vitro* was used to dissociate liver tissue (Kitani et al., 2011; Mo et al., 2020). For isolation of Kupffer cells, the livers of anesthetized mice were perfused as described above. After resuspension of the liver homogenate with 10 ml RPMI 1640 and centrifuged at 300×*g* for 5 min at 4 °C, the top aqueous phase was discarded, and the cell sediments was reserved. And then, cell sediments were resuspended with 10 ml RPMI 1640 and centrifuged at 50×*g* for 3 min at 4 °C. The top aqueous phase (cleared cell suspension) was transferred into a new 10 ml centrifuge tube and centrifuged at 300×*g* for 5 min at 4 °C, the top aqueous phase was discarded, and the cell sediments were reserved. The cell sediments mainly contained non-parenchymal cells of the liver that were KCs, sinusoidal endothelial cells and satellite cells. To purify the obtained cell population further, the method of selective adherence to plastic was used (Li et al., 2014). The cells were then seeded into 6-well plate at a density of 1–3 × 10^7^/well in Dulbecco’s Modified Eagle’s Medium (DMEM; Hyclone, Logan, UT, USA) supplemented with 10% fetal bovine serum (FBS; Hyclone, Logan, UT, USA) and 100 U/ml Penicillin/Streptomycin (Sigma, St. Louis, MI, USA) and incubated for 2 h in a 5% CO2 atmosphere at 37 °C. Non-adherent cells were then removed from the dish by gently washing with PBS, the adherent cells were KCs.

### Histology

Liver tissues from median and left lobes were collected, and embedded in paraffin according to standard histological procedures. Tissue sections were prepared and subjected to H&E staining for observation under a light microscope. To evaluate AIH inflammation, an inflammation score was performed as previously described (Bulau et al., 2011). For lobular inflammation, no inflammation was counted as 0, mild lobular inflammation (<10% of liver parenchyma) as 1, moderate lobular inflammation (10–50% of liver parenchyma) as 2, and a score of 3 was given for severe lobular inflammation (>50% of liver parenchyma). For portal inflammation, no portal inflammation was counted as 0, mild portal inflammation (<1/3 of portal tracts) as 1, moderate portal inflammation (approximately 1/2 of portal tracts) as 2, and a score of 3 was given for severe portal inflammation (>2/3 of portal tracts). The scores for portal and lobular inflammation were added, representing the AIH inflammation score.

### Analysis of liver fibrosis

The liver tissues were fixed in 10% neutral-buffered formalin, embedded in paraffin, sectioned at 5-μm thickness. Picrosirius staining was performed for detection of fibrosis. For quantitative assessment of fibrosis, a fibrosis score was performed as described previously (Deng et al., 2013). Briefly, a fibrosis score was performed by using a 0–4 scale: 0, no fibrosis; 1, minimal portal fibrosis; 2, portal fibrosis with septa formation; 3, localized bridging fibrosis; and 4, extensive bridging fibrosis.

### Real-time RT-PCR

The quantification of the expression of genes was performed using real-time RT-PCR. The sequences of the primers used for detecting gene expression were as follows:

**Table:** 

Gene name	Sense	Antisense
*Il1b* (IL-1β)	5′-TGGACCTTCCAGGATGAGGACA-3′	5′-GTTCATCTCGGAGCCTGTAGTG-3′
*Il6* (IL-6)	5′-CTGCAAGAGAC TTCCATCCAG-3′	5′-AGTGGTATAGACAGGTCTGTTGG-3′
*Tnfa* (TNF-α)	5′-CAGGCGGTGCCTATGTCTC-3′	5′-CGATCACCCCG AAGTTCAGTAG-3′
*Col1a1*	5′-ATGGATTCCCGTTCGAGTACG-3′	5′-TCAGCTGGATAGCGACATCG-3′
*Col1a2*	5′-CACCCCAGCG AAGAACTCATA-3′	5′-GCCACCATTGATAGTCTCTCCTAAC-3′
Il12 (IL-12)	5′-CCAGGTGTCTTAGCCAGTCC-3′	5′-GCAGTGCAG GAATAATGTTTCA-3′
Ifng (IFN-γ)	5′-ATGAACGCTACACACTGCATC-3′	5′-CCATCCTTTTGCCAGTTCCTC-3′
*Il4* (IL-4)	5′-GGTCTCAAC CCCCAGCTAGT-3′	5′-GCCGATGATCTCTCTCAAGTGAT-3′
*Il13* (IL-13)	5′-CCTGGCTCTTGCTTGCCTT-3′	5′-GGTCTTGTGTGATGTTGC TCA-3′
*TLR2*	5′-TTGCGTTACATCTTGGA ACTG-3′	5′-ACTACGTCTGACTCCGAGGG-3′
*TLR4*	5′-CTTCATTCAAGACCAAGCCTTTC-3′	5′-AACCGATGGACGTG TAAACCAG-3′
*iNOS*	5′-GAAGAAAACCCC TTGTGCTG-3′	5′-TCCAGGGATTCTGGAAC ATT-3′
*Arg-1*	5′-CTCCAAGCCAAAGTCCTTAGAG-3′	5′-AGG AGCTGTCATTAGGGACATC-3′
*Ym-1*	5′-CAGGTCTGGCAATTCTT CTG AA-3′	5′-GTCTTGCTCATGTGTGTAAGTGA-3′
*Il10 (IL-10)*	5′-ATCGATTTCTCCCCTGTGAA-3′	5′-TGTCAAATTCATTCATGGC CT-3′
*TGFB1 (TGF-β1)*	5′-CTCCCGTGGCTTCTAGTGC-3′	5′-GCCTTAGTTTGGACAGGATCTG-3′
*GAPDH*	5′-TCGTCCCGTAGACAA AATGG-3′	5′-TTGAGGTCAATGAAGGGGTC-3′

For sample analysis, the threshold was set based on the exponential phase of products, and C_T_ value for samples was determined. The resulting data were analyzed with the comparative C_T_ method for relative gene expression quantification against house keeping gene *GAPDH* (Yan et al., 2013).

### Immunohistochemistry

Liver tissue sections were prepared and subjected to immunohistochemical analysis as described earlier (Kitani et al., 2011). Anti-rabbit F4/80, Anti-rabbit CD86 and anti-rabbit CD163 (servicebio biotechnology, Wuhan, China) were used as primary Abs for detecting Kupffer cells, M1 phenotype macrophages and M2 phenotype macrophages, respectively. HRP conjugated secondary Ab was used for detecting macrophages, M1 macrophages and M2 macrophages in the liver tissues.

### Statistical analysis

All experiments were performed in triplicate and repeated three times, including three biological replicates and three technical replicates. The results were expressed as mean values ± SD and interpreted by using one-way analysis of variance (ANOVA). The differences were considered statistically significant when *P* < 0.05.

## Results

### Liver resident macrophages are involved in AIH chronic inflammatory processes

**Figure 1:**
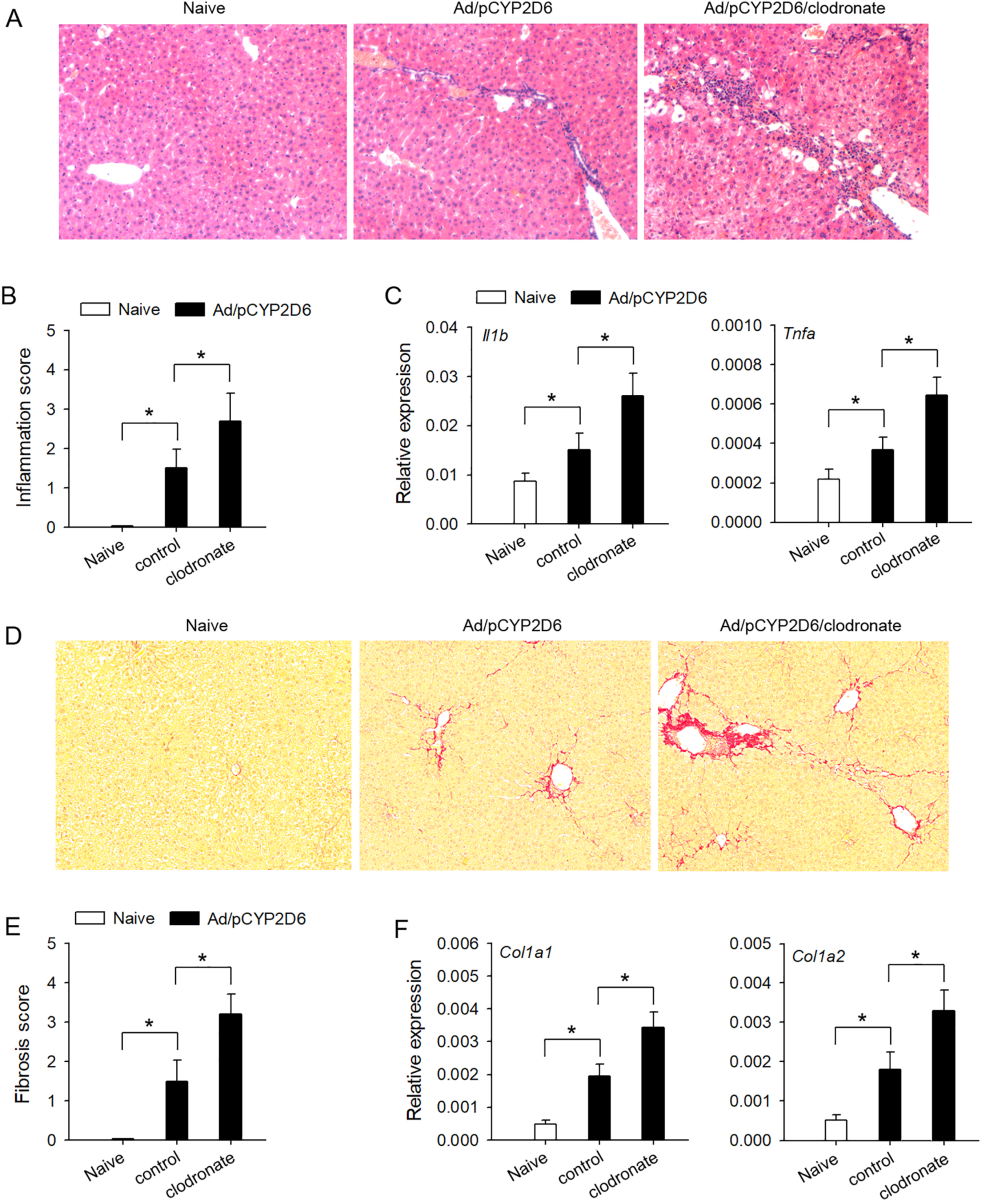
The effect of liver resident macrophages on AIH development. The mice (n = 6 per group) were untreated or received the injection of Ad/pCYP2D6 with or without the further injection of clodronate liposomes (see Fig. S1A). (A and B) On d30, slices of liver tissues (n=6 per group) were prepared and subjected to H&E staining for observing the inflammation. Representative images were shown (magnification 200×) (A) and inflammation scores were calculated (B). (C) On d30, the expression of *Il1b* and *Tnfa* gene in the liver was detected at mRNA level by real-time RT-PCR. (D and E) On d50, Picrosirius staining was used to detect liver fibrosis. Representative images were shown (magnification 200×) (D), and fibrosis scores were calculated (E). (F) The expression of *Col1a1* and *Col1a2* genes in the liver was detected by real-time RT-PCR on d50. *P < 0.05.

In order to explore the effect of liver resident macrophages on the liver inflammation in AIH, the clodronate liposomes were used to eliminate liver resident macrophages (Figs. S1A and S1B) based on our previous AIH mouse models (Chi et al., 2018). The lymphocyte infiltration was significantly increased by decreasing liver resident macrophages (Figs. 1A and 1B), and AIH inflammatory response, evaluated by the expression of *Il1b* and *Tnfa* genes in the liver, was also effectively increased by macrophage clearance (Fig. 1C). Further studies are needed to explore whether liver resident macrophages could effectively delay the development of liver fibrosis in AIH mice. The liver fibrosis induced by sustained AIH inflammation became more severe after liver resident macrophages depletion, evaluated by Picrosirius staining (Figs. 1D and 1E). Same results were observed when the expression of *Col1a1* and *Col1a2* genes in the liver was also detected (Fig. 1F). These results indicated that liver resident macrophages could effectively delay the development of AIH.

### The intensity of immune response is regulated by liver resident macrophages

**Figure 2:**
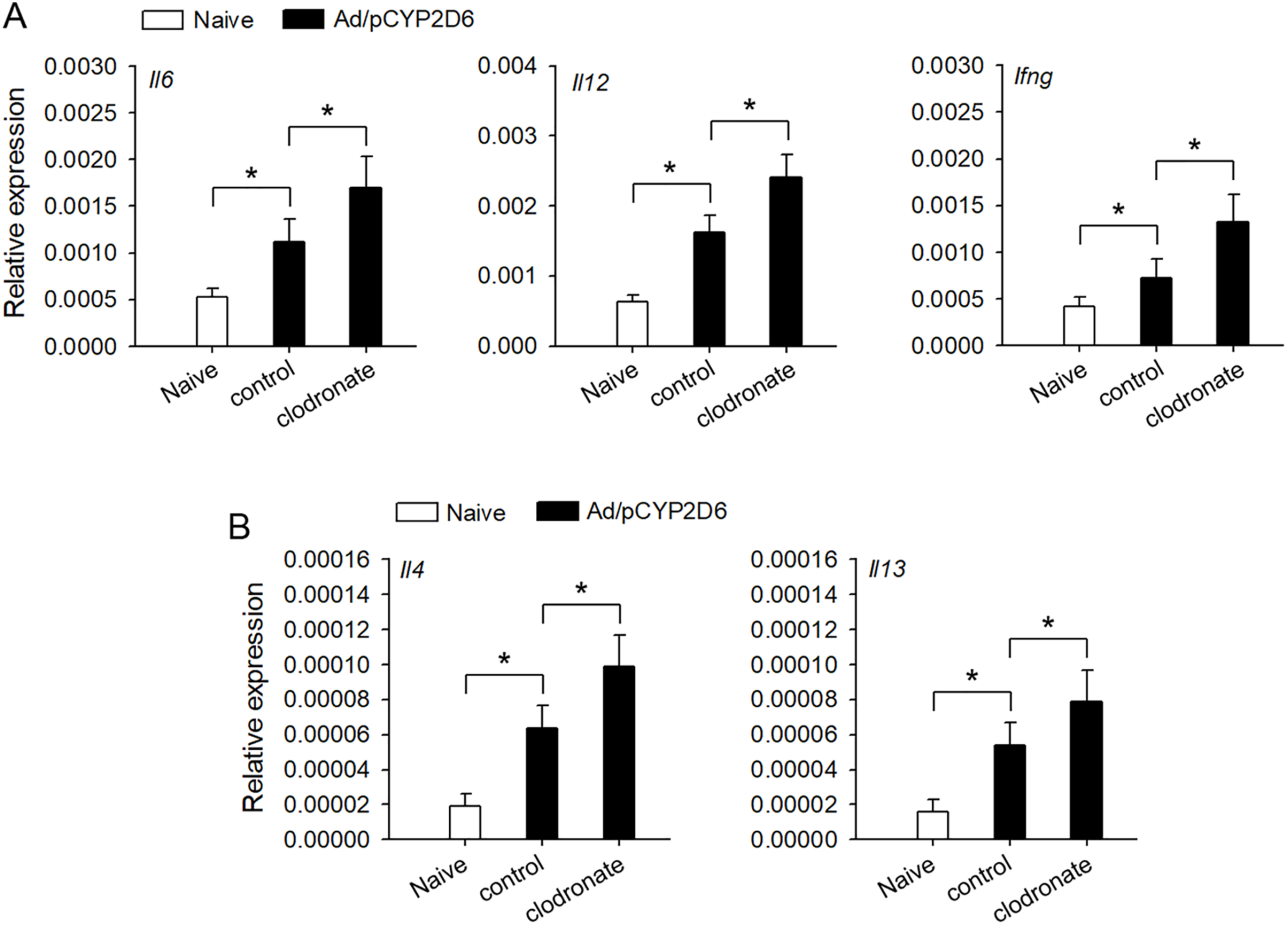
The effect of liver resident macrophages on the intensity of immune response in AIH. The mice were untreated or received the injection of Ad/pCYP2D6 with or without further injection of clodronate liposomes as indicated (see Fig. S1A). (A) On d30, the expression of *Il6*, *Il12* and Ifng genes was detected by real-time RT-PCR. (B) On d30, the expression of *Il4* and *Il13* genes was detected by real-time RT-PCR. *P < 0.05.

It is urgently needed to confirm whether liver resident macrophages could involve in the regulation of autoimmune response in AIH. Liver resident macrophages depletion could increase the intrahepatic expression of *IL-6* and *IL-12* that promote *IFN-γ* production, which favors Th1 response (Fig. 2A). Furthermore, *IL-4* and *IL-13* expression was also elevated to favor Th2 responses in AIH mice with liver resident macrophages depletion (Fig. 2B). These results suggested that liver resident macrophages could limit the intensity of autoimmune response, resulting in progressive and chronic AIH development.

### The effect TLR2 and TLR4 ligands on liver resident macrophages polarization in AIH development

**Figure 3:**
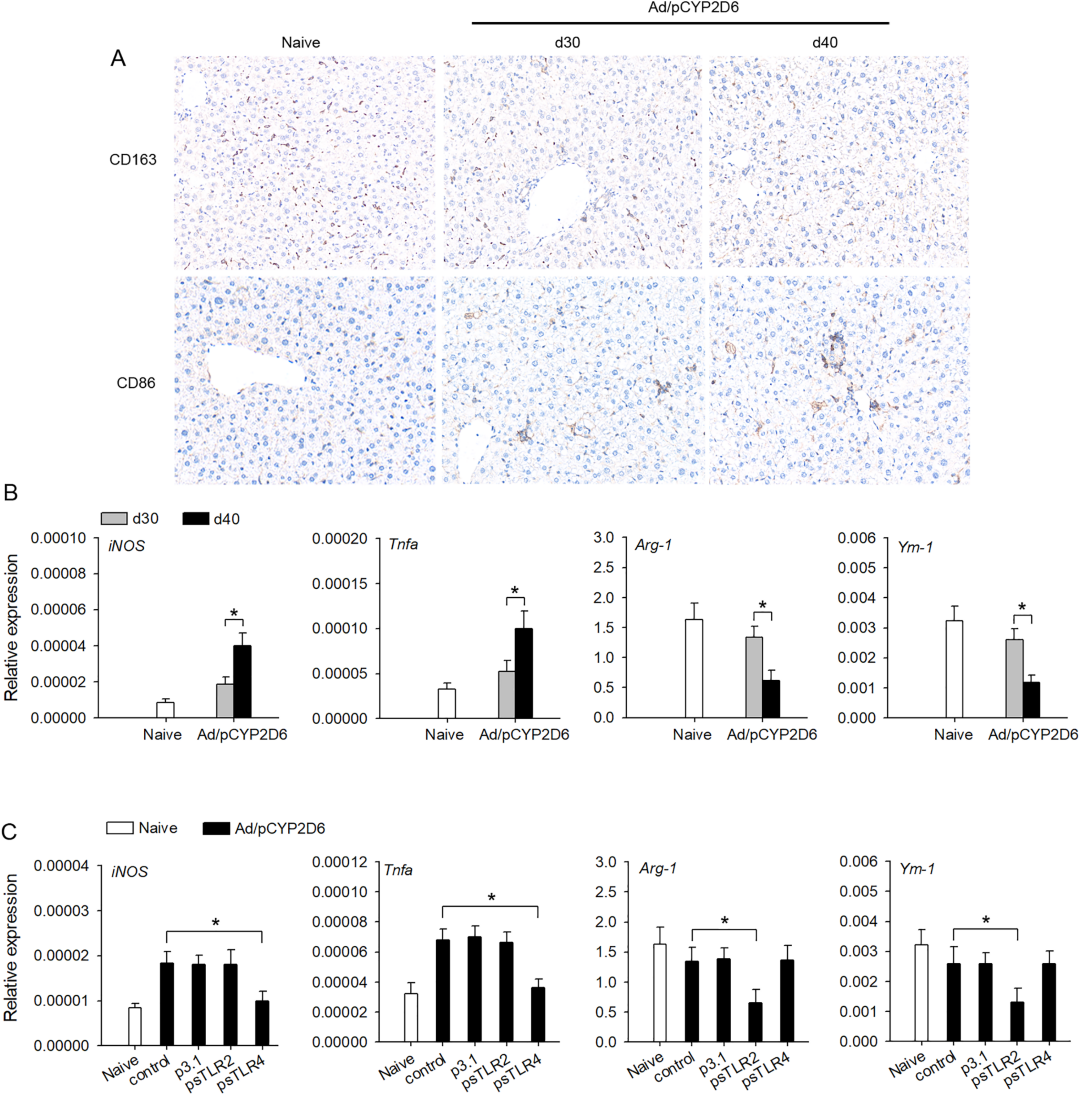
TLR2 and TLR4 ligands induce liver resident macrophages polarization in AIH. (A and B) The mice received the injection of Ad/pCYP2D6 at the indicated time points to induce AIH (See Fig. S2A). On d30 and d40, CD163+ macrophages and CD86+ macrophages in liver tissues of AIH mice were examined by immunohistochemistry (magnification 200×) (A). On d30 and d40, the mRNAs of M1 (iNOS and TNF-a) and M2 (Arg-1 and Ym-1) markers of liver tissues were tested by real-time RT-PCR (B). (C) The mice were untreated or received the injection of Ad/pCYP2D6 with or without further injection of p3.1, psTLR2 or psTLR4 as indicated (see Fig. S2B). On d30, the mRNAs of M1 and M2 markers of liver tissues were tested by real-time RT-PCR. *P < 0.05.

The effect of TLR2 or TLR4 ligands on liver macrophage polarization was further identified *in vivo* to better understand the underlying mechanisms of chronic inflammatory processes in AIH. We firstly detected the macrophage polarization marker in liver tissue by immunohistochemistry. The expression of CD163 (M2 marker) was decreased slowly in liver tissue of AIH mice. However, the expression of CD86 (M1 marker) was increased slowly in liver tissue during AIH development (Fig. 3A). Furthermore, the expression of M1 macrophage marker (*iNOS* and *TNF-a*) was markedly upregulated during AIH development. However, the expression of M2 macrophage marker (*Arg-1* and *Ym-1*) was significantly downregulated in liver tissue during AIH development (Fig. 3B). To further determine the effect TLR2/4 ligands on liver resident macrophages polarization, we expressed sTLR2 and sTLR4 to block TLR2 and TLR4 ligands (Yan et al., 2013; Li et al., 2017). In AIH mice, blocking TLR2 ligands resulted in M2 macrophage marker was significantly downregulated while blocking TLR4 ligands resulted in M1 macrophage marker was dramatically downregulated (Fig. 3C). These results indicated that TLR2 and TLR4 ligands might regulate liver macrophage polarization to influence AIH development.

### Changes in the cytokine secretion profile of liver resident macrophages following TLR2 and TLR4 ligand exposure and activation *in vitro*

**Figure 4:**
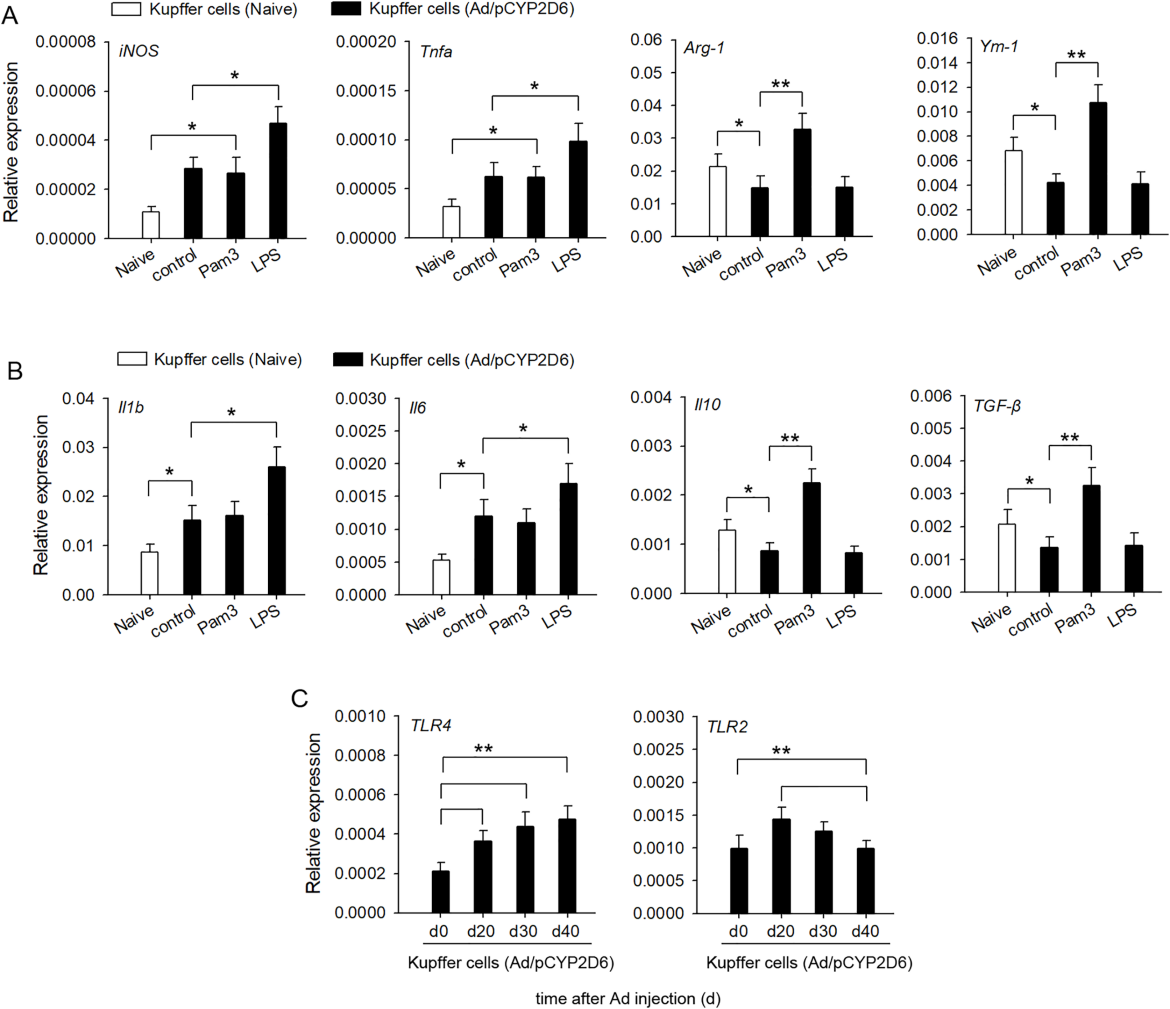
Changes in cytokine secretion profile of liver macrophages following TLR2 and TLR4 ligand exposure. The mice received the injection of Ad/pCYP2D6 at the indicated time points to induce AIH (See Fig. S2A). (A and B) Kupffer cells were isolated from AIH mice on d30 and stimulated for 24 h with 300 ng/ml Pam3, 200 ng/ml LPS. Change in gene expression of M1 and M2 markers of Kupffer cells was measured by real-time RT-PCR (A). In Kupffer cells, changes in expression of anti-inflammatory and pro-inflammatory genes were detected (B). (C) Kupffer cells were isolated from AIH mice at the indicated time points. The expression of TLR2 and TLR4 in Kupffer cells was detected at the mRNA level by real-time RT-PCR. *P < 0.05, **P < 0.01.

In order to confirm the involvement of TLR2 and TLR4 ligands in liver macrophage polarization and cytokine secretion by liver resident macrophages *in vitro*, Kupffer cells were isolated from liver tissues of AIH mice. The activation of TLR4 with LPS led to a significant upregulation of M1 macrophage markers, whereas expression of M2 macrophage markers was no significant difference compared with the control group (Fig. 4A). In contrast, TLR2 stimulation with Pam3 significantly upregulated M2 macrophage markers, whereas expression of M1 macrophage markers showed no significant change compared with control group (Fig. 4A). The stimulation of Kupffer cells with LPS, but not with Pam3, strongly induced the production of proinflammatory cytokines *IL-1β* and *IL-6* (Fig. 4B). In contrast, the stimulation of Kupffer cells with Pam3, but not with LPS, strongly induced the production of anti-inflammatory cytokines *IL-10* and *TGF-β* (Fig. 4B). TLR2 expression increased first and then decreased in AIH development. By contrast, TLR4 remained highly expressed in AIH development (Fig. 4C). These results indicated that TLR4 ligand could promote M1 Kupffer cells producing proinflammatory cytokines, whereas TLR2 ligand could promote M2 Kupffer cells producing anti-inflammatory cytokines.

## Discussion

On the one hand, the activated liver resident macrophages are present in portal infiltrates and play a crucial role in the inflammatory response in AIH, and on the other hand, the defective function of liver resident macrophages may be involved in the pathogenesis of AIH (Zhang et al., 2018b; Lin et al., 2016). This study showed that liver resident macrophages could effectively delay the development of AIH and limit the intensity of the autoimmune response, resulting in chronic progressive AIH. One potential reason for this result may be that TLR2 and TLR4 ligands regulate macrophage polarization to promote autoimmune hepatitis progression.

The dynamic changes in the activation of Kupffer cells should be closely linked to the hepatic inflammatory response as a resident macrophage. Kupffer cells alleviated liver damage and induced hepatocyte survival and regeneration (Zheng et al., 2017). M2-like Kupffer cells may protect against acute liver injury, thereby suppressing pro-inflammatory responses and attenuating liver injury (Kono, Onda & Yanagida, 2014). Additional studies showed that depletion or functional inhibition of macrophages exhibits promising effects in attenuating the severity of several autoimmune diseases (Ponomarev et al., 2011; Xiao et al., 2013; Zhang et al., 2018b). However, this study showed that M2 Kupffer cells were the dominant phenotype of macrophages. Hepatic inflammation was progressively aggravated after depletion of these Kupffer cells during AIH immune response stage. The hepatic fibrosis and the intensity of Th1 and Th2 response were more severe after these macrophages depletion. It had been reported that Th1 response could be associated with the induction of cellular immunity by secreting cytokines whereas Th2 response could control humoral immune response by mediating B-cell proliferation, differentiation and specific antibodies production (Zhao et al., 2018). Macrophage production was regulated by Th1 and Th2 cytokines. The maintenance of M2 macrophages was thought to require IL-4-producing Th2 cells and switch from M2 to M1 macrophages may be triggered by Th1 environment (Wan et al., 2015). Thus, the presence of high numbers of M2 Kupffer cells during early stage of immune responses might effectively delay the development of AIH to drive ongoing inflammation development. The number of M1 and M2 macrophages may depend on the degree of chronic inflammatory reaction, which needs to be further investigated in the future. Previous studies showed that chronic inflammatory responses may require the presence of resident tissue macrophages even though recruited macrophages were generally more prominent in chronic inflammatory conditions than in acute ones (Toh et al., 2014; Hamilton, Cook & Tak, 2016).

Previous studies indicated that dysfunction of macrophages was critical in the pathogenesis of numerous autoimmune diseases. The macrophage functions depend on their polarizing microenvironment and controlling their responses was a key factor for the outcome of both inflammation and autoimmune disease (Arnold et al., 2014). Resident macrophages in rheumatoid arthritis (RA) exhibited not only M1-like proinflammatory activity but also M2-like anti-inflammatory activity, which showed that macrophages display a remarkable plasticity (Soler et al., 2015; Malyshev & Malyshev, 2015). The total and M1 macrophage populations were increased in AIH patients, whereas M2 macrophage population decreased (Wang et al., 2019). Persistent and excessive activation of macrophages results in pathological inflammation and fibrosis in chronic liver diseases. The cytokine production from macrophages is essential for initiating inflammatory response, which led to the breaking of immune microenvironment balance, shifting the local environment toward a proinflammatory state and resulting in tissue damage (Goverman, 2011; Bartneck et al., 2016). This study showed that Kupffer cells progressively polarized from M2 to M1 during AIH development, causing a decrease in the expression of anti-inflammatory cytokines and an increase in pro-inflammatory cytokines although M2 Kupffer cells were dominant at early stages of immune responses. IFN-γ production by CD4+Th1 cells promoted M1 macrophage polarization, indicating a positive feedback loop in M1 macrophage polarization. The proinflammatory chemokines produced by M1 macrophages could further promote macrophage infiltration (Zhang et al., 2021; Toba et al., 2015).

Macrophages exhibit a high expression of pattern recognition receptors, especially Toll-like receptors (TLRs), whose interaction with agonists triggered cell activation (Freitas et al., 2016). TLR2/4 expression was increased in peripheral blood mononuclear cells and monocytes from patients with a variety of autoimmune diseases, including type 1 diabetes, RA and SLE (Hamerman et al., 2016; Ren et al., 2016). TLR2/4 signaling could amplify joint inflammation in macrophages in RA (Goh & Midwood, 2012). TLR2 agonist PAM3 similarly induced monocytes from Lupus patients to preferentially differentiate into M2-like macrophages, which represented a novel approach to the therapy of SLE (Horuluoglu et al., 2019). TLR4 ligand LPS polarize monocytes towarded classically activated M1 macrophages, which produced pro-inflammation cytokines. TLR4 signaling and inflammatory cytokine could serve as a useful therapeutic target for treatment of AIH (Zhang et al., 2016). It is likely that a reduced frequency of anti-inflammatory M2 macrophages or a prolonged activation of M1 macrophages could be implicated in the development of detrimental inflammation and autoimmunity (Funes et al., 2018). This study showed that TLR2 ligand could induce M2 Kupffer cells and promote production of anti-inflammatory cytokines, whereas TLR4 ligand could induce M1 Kupffer cells and promote production of proinflammatory cytokines at AIH immune response stage. Interestingly, TLR2 expression increased first and then decreased, whereas TLR4 expression remained elevated in Kupffer cells during AIH development. Changes in TLR2 and TLR4 ligands successively increased TLR4 expression and decreased TLR2 expression, further polarizing Kupffer cells from M2 to M1, although M2 Kupffer cells were the predominant phenotype of macrophages at early stages of immune responses.

In conclusion, this study showed that TLR2 and TLR4 ligands could regulate liver resident macrophages polarization to favor chronic autoimmune hepatitis development. M2 macrophages were dominant during early stages of immune responses, while TLR4 continued to express highly and expression of TLR2 was decreased, which would resulted in polarization of liver resident macrophages from M2 to M1 to promote the development of chronic autoimmune hepatitis. Therefore, it may be an effective and promising therapeutic strategy by manipulating the macrophage polarization and inhibiting early activation of M1 macrophage in AIH development.

## Supplemental Information

10.7717/peerj.14871/supp-1 Supplemental Information 1 Raw data: inflammation and fibrosis scores and qPCR data.

10.7717/peerj.14871/supp-2 Supplemental Information 2 Supplementary data.

10.7717/peerj.14871/supp-3 Supplemental Information 3 ARRIVE 2.0 checklist.

## Additional Information and Declarations

### Data Availability

The following information was supplied regarding data availability: The qPCR data is available in the Supplemental File.

## References

[ref-1] Amin MA, Haas CS, Zhu K, Mansfield PJ, Kim MJ, Lackowski NP, Koch AE (2006). Migration inhibitory factor up-regulates vascular cell adhesion molecule-1 and intercellular adhesion molecule-1 via Src, PI3 kinase, and NFkappaB. Blood.

[ref-2] Arnold CE, Whyte CS, Gordon P, Barker RN, Rees AJ, Wilson HM (2014). A critical role for suppressor of cytokine signalling 3 in promoting M1 macrophage activation and function *in vitro* and *in vivo*. Immunology.

[ref-3] Arterbery AS, Yao J, Ling A, Avitzur Y, Martinez MLS, Deng YH, Geliang G, Mehta S, Wang GL, Knight J, Ekong UD (2018). viaInflammasome priming mediated toll-like receptors 2 and 4, induces Th1-like regulatory T cells in autoimmune hepatitis. Frontiers in Immunology.

[ref-4] Bartneck M, Fech V, Ehling J, Govaere O, Warzecha KT, Hittatiya K, Vucur M, Gautheron J, Luedde T, Trautwein C, Lammers T, Roskams T, Jahnen-Dechent W, Tacke F (2016). Histidine-rich glycoprotein promotes macrophage activation and inflammation in chronic liver disease. Hepatology.

[ref-5] Boyman O, Purton JF, Surh CD, Sprent J (2007). Cytokines and T-cell homeostasis. Current Opinion in Immunology.

[ref-6] Bulau AM, Fink M, Maucksch C, Kappler R, Mayr D, Wagner K, Bufler P (2011). In vivo expression of interleukin-37 reduces local and systemic inflammation in concanavalin A-induced hepatitis. Scientific World Journal.

[ref-7] Burdo TH, Lackner A, Williams KC (2013). Monocyte/macrophages and their role in HIV neuropathogenesis. Immunological Reviews.

[ref-8] Chi G, Feng YX, Ru YX, Xiong T, Gao Y, Wang H, Luo ZL, Mo R, Guo F, He YP, Zhang GM, Tian DA, Feng ZH (2018). TLR2/4 ligand-amplified liver inflammation promotes initiation of autoimmune hepatitis due to sustained IL-6/IL-12/IL-4/IL-25 expression. Molecular Immunology.

[ref-9] Chi G, Pei JH, Ma QY, Ru YX, Feng ZH (2020). Chemical induced inflammation of the liver breaks tolerance and results in autoimmune hepatitis in Balb/c mice. Immunology Letters.

[ref-10] Czaja AJ (2015). Adoptive cell transfer in autoimmune hepatitis. Expert Review of Gastroenterology & Hepatology.

[ref-11] Deng YR, Ma HD, Tsuneyama K, Yang W, Wang YH, Lu FT, Liu CH, Liu P, He XS, Diehl AM, Gershwin ME, Lian ZX (2013). STAT3-mediated attenuation of CCl4-induced mouse liver fibrosis by the protein kinase inhibitor sorafenib. Journal of Autoimmunity.

[ref-12] Freitas MS, Oliveira AF, da Silva TA, Fernandes FF, Gonçales RA, Almeida F, Roque-Barreira MC (2016). Paracoccin induces M1 polarization of macrophages via interaction with TLR4. Frontiers in Microbiology.

[ref-13] Funes SC, Rios M, Escobar VJ, Kalergis AM (2018). Implications of macrophage polarization in autoimmunity. Immunology.

[ref-14] Geng H, Zhang GM, Li D, Zhang H, Yuan Y, Zhu HG, Xiao H, Han LF, Feng ZH (2006). Soluble form of T cell Ig mucin 3 is an inhibitory molecule in T cell-mediated immune response. Journal of Immunology.

[ref-15] Goh FG, Midwood KS (2012). Intrinsic danger: activation of toll-like receptors in rheumatoid arthritis. Rheumatology.

[ref-16] Goverman JM (2011). Immune tolerance in multiple sclerosis. Immunological Reviews.

[ref-17] Hamerman JA, Pottle J, Ni MJ, He YT, Zhang ZY, Buckner JH (2016). Negative regulation of TLR signaling in myeloid cells--implications for autoimmune diseases. Immunological Reviews.

[ref-18] Hamilton JA, Cook AD, Tak PP (2016). Anti-colony-stimulating factor therapies for inflammatory and autoimmune diseases. Nature Reviews Drug Discovery.

[ref-19] Horuluoglu B, Bayik D, Kayraklioglu N, Goguet E, Kaplan MJ, Klinman DM (2019). PAM3 supports the generation of M2-like macrophages from lupus patient monocytes and improves disease outcome in murine lupus. Journal of Autoimmunity.

[ref-20] Ikeda A, Aoki N, Kido M, Iwamoto S, Nishiura H, Maruoka R, Chiba T, Watanabe N (2014). Progression of autoimmune hepatitis is mediated by IL-18-producing dendritic cells and hepatic CXCL9 expression in mice. Hepatology.

[ref-21] Kitani H, Takenouchi T, Sato M, Yoshioka M, Yamanaka N (2011). A simple and efficient method to isolate macrophages from mixed primary cultures of adult liver cells. Journal of Visualized Experiments.

[ref-22] Kono H, Onda A, Yanagida T (2014). Molecular determinants of sterile inflammation. Current Opinion in Immunology.

[ref-23] Li PZ, Li JZ, Li M, Gong JP, He K (2014). An efficient method to isolate and culture mouse Kupffer cells. Immunology Letters.

[ref-24] Li YC, Zou JM, Luo C, Shu Y, Luo J, Qin J, Wang Y, Li D, Wang SS, Chi G, Guo F, Zhang GM, Feng ZH (2017). Circulating tumor cells promote the metastatic colonization of disseminated carcinoma cells by inducing systemic inflammation. Oncotarget.

[ref-25] Lin R, Zhang J, Zhou L, Wang BM (2016). Altered function of monocytes/macrophages in patients with autoimmune hepatitis. Molecular Medicine Reports.

[ref-26] Liu Y, Liu H, Zhu JS, Bian ZL (2019). Interleukin-34 drives macrophage polarization to the M2 phenotype in autoimmune hepatitis. Pathology Research and Practice.

[ref-27] Malyshev I, Malyshev Y (2015). Current concept and update of the macrophage plasticity concept: intracellular mechanisms of reprogramming and M3 macrophage switch phenotype. Biomed Research International.

[ref-28] Mieli-Vergani G, Vergani D, Czaja A, Manns MP, Krawitt EL, Vierling JM, Lohse AW, Montano-Loza AJ (2018). Autoimmune hepatitis. Nature Reviews Disease Primers.

[ref-29] Mo R, Feng XX, Wu YN, Wang H, He YP, Sun H-H, Guo F, Chen Q, Yan W, Li PY, Liu M, Zhang GM, Tian DA, Feng ZH (2020). Hepatocytes paradoxically affect intrahepatic IFN-γ production in autoimmune hepatitis due to Gal-9 expression and TLR2/4 ligand release. Molecular Immunology.

[ref-30] National Research Council of the National Academics (1996). Guide for the Care and Use of Laboratory Animals.

[ref-31] Park YJ, Liu G, Tsuruta Y, Lorne E, Abraham E (2009). Participation of the urokinase receptor in neutrophil efferocytosis. Blood.

[ref-32] Ponomarev ED, Veremeyko T, Barteneva N, Krichevsky AM, Weiner H (2011). MicroRNA-124 promotes microglia quiescence and suppresses EAE by deactivating macrophages via the C/EBP-α-PU.1 pathway. Nature Medicine.

[ref-33] Quero L, Hanser E, Manigold T, Tiaden AN, Kyburz D (2017). TLR2 stimulation impairs anti-inflammatory activity of M2-like macrophages, generating a chimeric M1/M2 phenotype. Arthritis Research & Therapy.

[ref-34] Ren CC, Zhang QX, de Haan BJ, Zhang H, Faas MM, de Vos P (2016). Identification of TLR2/TLR6 signalling lactic acid bacteria for supporting immune regulation. Scientific Reports.

[ref-35] Schultheiß C, Simnica D, Willscher E, Oberle A, Fanchi L, Bonzanni N, Wildner NH, Schulze ZWJ, Weiler-Normann C, Lohse AW, Binder M (2021). Next-generation immunosequencing reveals pathological T-cell architecture in autoimmune hepatitis. Hepatology.

[ref-36] Soler PB, Estrada CL, Izquierdo E, Criado G, Nieto C, Municio C, González-Alvaro I, Sánchez-Mateos P, Pablos JL, Corbí AL, Puig-Kröger A (2015). Macrophages from the synovium of active rheumatoid arthritis exhibit an activin A-dependent pro-inflammatory profile. Journal of Pathology.

[ref-37] Stone AEL, Green R, Wilkins C, Hemann EA, Gale M (2019). RIG-I-like receptors direct inflammatory macrophage polarization against West Nile virus infection. Nature Communications.

[ref-38] Terziroli Beretta-Piccoli B, Mieli-Vergani G, Vergani D (2022). Autoimmmune hepatitis. Cellular & Molecular Immunology.

[ref-39] Toba H, de Castro Brás LE, Baicu CF, Zile MR, Lindsey ML, Bradshaw AD (2015). Secreted protein acidic and rich in cysteine facilitates age-related cardiac inflammation and macrophage M1 polarization. American Journal of Physiology-Cell Physiology.

[ref-40] Toh ML, Bonnefoy JY, Accart N, Cochin S, Pohle S, Haegel H, De MM, Zemmour C, Preville X, Guillen C, Thioudellet C, Ancian P, Lux A, Sehnert B, Nimmerjahn F, Voll RE, Schett GEE (2014). Bone- and cartilage-protective effects of a monoclonal antibody against colony-stimulating factor 1 receptor in experimental arthritis. Arthritis & Rheumatology.

[ref-41] Wan WZ, Liu Q, Lionakis MS, Marino APMP, Anderson SA, Swamydas M, Murphy PM (2015). Atypical chemokine receptor 1 deficiency reduces atherogenesis in ApoE-knockout mice. Cardiovascular Research.

[ref-42] Wang YJ, Guo XP, Jiao GH, Luo LL, Zhou L, Zhang J, Wang BM (2019). Splenectomy promotes macrophage polarization in a mouse model of concanavalin A- (ConA-) induced liver fibrosis. Biomed Research International.

[ref-43] Wu JL, Zou JY, Hu ED, Chen DZ, Chen L, Lu FB, Xu LM, Zheng MH, Li H, Huang Y, Jin XY, Gong YW, Lin Z, Wang XD, Zhao MF, Chen YP (2017). Sodium butyrate ameliorates S100/FCA-induced autoimmune hepatitis through regulation of intestinal tight junction and toll-like receptor 4 signaling pathway. Immunology Letters.

[ref-44] Xiao Y, Jin J, Chang M, Chang JH, Hu HB, Zhou XF, Brittain GC, Stansberg C, Torkildsen Ø, Wang XD, Brink R, Cheng XH, Sun SC (2013). Peli1 promotes microglia-mediated CNS inflammation by regulating Traf3 degradation. Nature Medicine.

[ref-45] Yan B, Wei JJ, Yuan Y, Sun R, Li D, Luo J, Liao SJ, Zhou YH, Shu Y, Wang Q, Zhang GM, Feng ZH (2013). IL-6 cooperates with G-CSF to induce protumor function of neutrophils in bone marrow by enhancing STAT3 activation. Journal of Immunology.

[ref-46] Zhang J, Guo LP, Liu MJ, Jing Y, Zhou SM, Li HX, Li YN, Zhao JW, Zhao XL, Karunaratna N, Jiang K, Zhou L, Wang BM (2018a). Receptor-interacting protein kinase 3 mediates macrophage/monocyte activation in autoimmune hepatitis and regulates interleukin-6 production. United European Gastroenterology Journal.

[ref-47] Zhang X, Wang Y, Yuan J, Li N, Pei S, Xu J, Luo X, Mao C, Liu J, Yu T, Gan S, Zheng Q, Liang Y, Guo W, Qiu J, Constantin G, Jin J, Qin J, Xiao Y (2018b). Macrophage/microglial Ezh2 facilitates autoimmune inflammation through inhibition of Socs3. Journal of Experimental Medicine.

[ref-48] Zhang QQ, Wang Y, Zhai NC, Song HX, Li HJ, Yang Y, Li TY, Guo XL, Chi BR, Niu JQ, Crispe IN, Su LS, Tu ZK (2016). HCV core protein inhibits polarization and activity of both M1 and M2 macrophages through the TLR2 signaling pathway. Scientific Reports.

[ref-49] Zhang LJ, Zhang K, Zhang JY, Zhu JR, Xi Q, Wang HF, Zhang ZM, Cheng YN, Yang GZ, Liu HK, Guo XD, Zhou DM, Xue ZY, Li Y, Zhang Q, Da YR, Liu L, Yin ZN, Yao Z, Zhang RX (2021). Loss of fragile site-associated tumor suppressor promotes antitumor immunity via macrophage polarization. Nature Communications.

[ref-50] Zhao XH, Ding S, Geng C, Man Z, Pan MZ, Sun LL, Hu BJ, Wang H (2018). Anti-CD200 attenuates concanavalin A induced hepatitis via modulating the imbalance of CD4 T lymphocyte differentiation in mice. American Journal of Translational Research.

[ref-51] Zhao SC, Wang C, Xu H, Wu WQ, Chu ZH, Ma LS, Zhang YD, Liu FD (2017). Age-related differences in interferon regulatory factor-4 and -5 signaling in ischemic brains of mice. Acta Pharmacologica Sinica.

[ref-52] Zheng QF, Bai L, Duan ZP, Han YP, Zheng SJ, Chen Y, Li JS (2017). M2-like Kupffer cells in fibrotic liver may protect against acute insult. World Journal of Gastroenterology.

