# Supplementary material for: The imbalance of liver resident macrophages polarization promotes chronic autoimmune hepatitis development in mice
Source: PeerJ. 2023 Feb 7;11:e14871. doi: 10.7717/peerj.14871 (PMC9912947; doi:10.7717/peerj.14871)
Supplement: Supplemental Information 2 [file peerj-11-14871-s002.pdf]

The imbalance of liver macrophages polarization promotes chronic autoimmune hepatitis development

Supplementary Figures

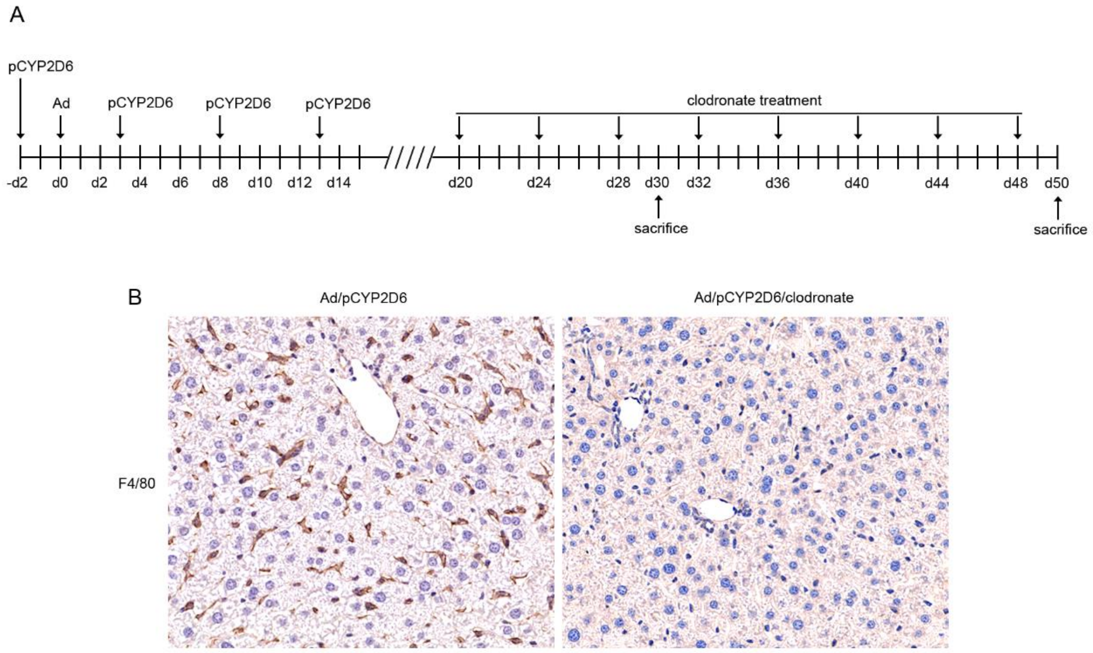

Supplementary Figure S1 Kupffer cells depletion in AIH mice. (A) The mice received the i.v. injection of adenovirus (Ad, 10^9 pfu) and plasmid pCYP2D6 (50 μg per injection) at the indicated time points to induce AIH. At the indicated time points, the mice received further i.p. injection of clodronate liposomes (100μl) every 4 days from d20 after AIH mice model establishment and the mice (n=6 per group) were sacrificed for the following experiments. **(B)** On d30, IHC analyses for F4/80 were performed to evaluate the effect of macrophage depletion by clodronate liposome.

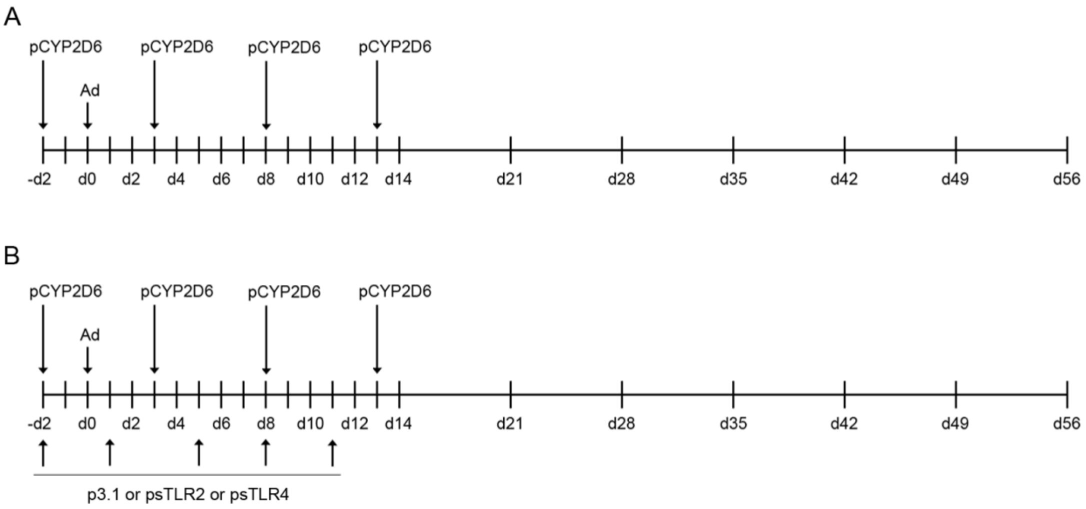

Supplementary Figure S2 Induction of AIH and treatment protocols for AIH mice. (A and B) The mice received the i.v. injection of adenovirus (Ad, 10^9 pfu) and plasmid pCYP2D6 (50 μg per injection) at the indicated time points to induce autoimmune hepatitis (A). At the indicated time points, the mice further received the i.v. injection of plasmid p3.1 (100ug per injection), psTLR2 (100ug per injection) or psTLR4 (100ug per injection) as described in Methods (B).
